# Replication of a Dog-Origin H6N1 Influenza Virus in Cell Culture and Mice

**DOI:** 10.3390/v12070704

**Published:** 2020-06-30

**Authors:** Shou-Kuan Tsai, Cheng-Hsin Shih, Hui-Wen Chang, Kuang-Huan Teng, Wei-En Hsu, Han-Jia Lin, Han-You Lin, Ching-Huei Huang, Hui-Wen Chen, Lih-Chiann Wang

**Affiliations:** 1 Department of Bioscience and Biotechnology, National Taiwan Ocean University, Keelung 20224, Taiwan; Eric.tsai@bioptic.com.tw (S.-K.T.); hanjia@mail.ntou.edu.tw (H.-J.L); 2 School of Veterinary Medicine, National Taiwan University, Taipei 10617, Taiwan; st86123@gmail.com (C.-H.S.); huiwenchang@ntu.edu.tw (H.-W.C.); tkuanghuan18408@gmail.com (K.-H.T.); hotaru510266@gmail.com (W.-E.H.); linhanyou@ntu.edu.tw (H.-Y.L.); winnichen@ntu.edu.tw (H.-W.C.); 3 Ten Giga Bio-Technology Co., Ltd., Keelung 20224, Taiwan; ilfu00501@gmail.com

**Keywords:** dog, H6N1, influenza A virus, interspecies

## Abstract

The world’s first natural avian-origin H6N1 influenza A virus infection case in dogs was confirmed in Taiwan in 2014. The H6N1 virus in chickens has been endemic in Taiwan since 1972. Whether the dog H6N1 virus has interspecies transmission potential is the key issue we aim to understand. Following one virus passage in embryonated eggs and two further passages in MDCK cells, we obtained two virus derivatives, E01EE (PB1 739E and PB2 627E) and E01GK (PB1 739G and PB2 627K), respectively. The pathogenicity of E01EE and E01GK was investigated using plaque assay, growth dynamic analysis and cell viability quantification in cells from different animal species. The impact of amino acid mutation on PB1 739 and PB2 627 on viral ribonucleoprotein (RNP) activity was also analyzed. Further mouse infection experiments were performed. The results showed that both E01EE and E01GK decreased cell relative viability of canine MDCK cells, human A549 cells and chicken DF1 cells. E01Gk caused greater cellular harm in MDCK and A549 cells and had significantly higher virus titers in all of the cells compared to E01EE. The PB2 627K but not PB1 739G was the critical mutation that influenced the viral RNP activity. Both E01EE and E01GK caused mice pneumonia and considerable virus shedding, especially E01GK. This report verifies PB2 E627K mutation in virulence and spotlights the potential for the dog H6N1 virus to extend interspecies transmission.

## 1. Introduction

The influenza A virus has a wide host range and causes varied disease symptoms, making it an important zoonotic pathogen in the world [1]. Interspecies transmission of equine influenza A(H3N8) virus was first identified in a respiratory disease outbreak occurring in greyhounds in Florida in 2004 [2]. In 2007 avian-origin H3N2 canine influenza A virus was also discovered and confirmed in South Korea [3]. Both H3N8 and H3N2 subtypes were able to cause sustained transmission among dogs [4,5]. Sporadic influenza infections in dogs were successively reported in Asia, such as H3N2, H3N1, H5N2 and H5N1 in China, South Korea and Thailand [6,7,8,9,10,11,12]. Avian influenza H6N1 viruses have been widespread in chickens in Taiwan since 1972 [13]. The world’s first H6N1 human infection and dog natural infection cases happened in Taiwan in 2013 [14] and 2014 [15], respectively. The human H6N1 virus (A/Taiwan/2/2013) and the dog H6N1 virus (A/canine/Taiwan/E01/2014) were demonstrated to originate from avian sources [15], which have attracted attention to their interspecies transmission roles. Following one virus passage in embryonated eggs and two further passages in MDCK cells, we obtained two virus derivatives of the dog H6N1 virus, respectively. These two derivatives were different in PB1 739 and PB2 627. PB1 and PB2 together with PA and NP proteins of influenza viruses constitute viral ribonucleoprotein (RNP) complex, taking charge of the viral transcription and replication [16]. Mutations on these RNP component proteins could change the viral polymerase activity and virulence, such as PA P190S and PA Q400P in H7N3 [17], PA T97I in H6N1 [18], PB1 T296R in H1N1 [19], PB2 E627K in H7N9 [20] and PB2 Q591K in H9N2 [21]. This study was aimed to analyze the dog H6N1 infectivity in different animal species and compared the characteristics of its two derivates. Mouse inoculation experiments provided us with a deeper understanding of the dog H6N1 virus in mammalian virulence. The dog H6N1 virus was found to have infectivity in mammals, especially when it possessed the PB2 E627K mutation. Viral pathogenicity was influenced by amino acid alteration via passages in different culture systems, which implied that the dog H6N1 virulence could be increased once it adapted to mammals.

## 2. Materials and Methods 

### 2.1. Viruses 

A/canine/Taiwan/E01/2014 (H6N1) (abbreviated as E01) was isolated from a dog nasal swab via inoculation of SPF embryonated eggs, possessing 739E (Glutamic acid) on PB1 protein and 627K (Lysine) on PB2 protein [15]. E01 was proliferated through one SPF embryonated egg passage and showed the PB2 627E mutation; this virus was termed as E01EE. E01EE was multiplied through two MDCK cell passages and its PB1 739 altered to G (Glycine) and PB2 627 turned back to K; this virus was termed as E01GK. E01EE and E01GK were then purified through three times of plaque purifications to ensure the fixed amino acids characteristic. Further whole genome sequencing was performed, and the amino acids were all the same except for PB1739 and PB2 627. The amino acid differences among these viruses are denoted in Table 1. E01 was propagated because of depletion. The progeny E01EE and E01GK were used instead of E01 in the following studies. 

### 2.2. Cells

Cells from three different animal origins were used to examine the cell type influence on virus adaptation, including Madin–Darby canine kidney (MDCK) cells, human lung epithelial carcinoma A549 cells and chicken embryo fibroblast DF-1 cells. All cells were purchased from American Type Culture Collection (ATCC, Manassas, VA, USA), and cultured in Dulbecco’s modified Eagle’s medium (DMEM) (Thermo Fisher Scientific, Waltham, MA, USA) with 1% penicillin/streptomycin/amphotericin antibiotic mixture (Thermo) and 10% fetal bovine serum (Thermo) at 37 °C in a humidified 5% CO2 atmosphere. While the cells were infected, they were cultured in virus growth medium (VGM) with 2 μg/mL, none or 0.05 μg/mL of TPCK-trypsin (Thermo) for MDCK, A-549 and DF-1 cell, respectively. The VGM contains DMEM (Thermo), 1% antibiotic mixture (Thermo), 0.2% BSA (Goldbio, Louis, MO, USA) and 25 mM HEPES buffer (Goldbio). 

### 2.3. Plaque Assay 

MDCK cells (1 × 10^6^ cells/well) incubated overnight in six-well tissue culture plates were seeded with 1 mL of a viral suspension which was serially diluted from 10^−2^ to 10^−6^ and incubated at 37 °C for 1 h. Afterwards, the cells were washed and incubated with overlay DMEM (Thermo), 2 μg/mL TPCK-trypsin (Thermo) and 0.8 % agarose at 37 °C for 4 days. Four percent paraformaldehyde (Thermo) was added to fix the agarose layer for 1 h. The agarose layer was removed using running tap water. The cell monolayer was stained with 0.5% crystal violet (Thermo) for 10 min. The plaques were counted, and the multiplicity of infection (MOI) was calculated. 

### 2.4. Viral Dynamics

MDCK, A549 and DF-1 cells (1 × 10^6^ cells/ well) were incubated overnight in six-well tissue culture plates. The confluent cells were then washed and absorbed with 0.01 MOI of E01EE and E01GK at 37 °C for 1 h. Cells without virus inoculation were used as a negative control. Following washing to remove the virus, the cells were incubated for another 96 h. The culture supernatants were collected at 24 h intervals and stored at −80 °C until use. The 50% tissue culture infectious dose (TCID_50_) of the collected supernatants mentioned above was determined on MDCK cells using WHO protocol [22].

### 2.5. MTT (3-(4,5-Dimethylthiazol-2-yl)-2,5-Diphenyltetrazolium Bromide) Assay

The infected cell viability was quantified using MTT assay. This colorimetric assay is based on the reduction of a yellow tetrazolium salt (3-(4,5-dimethylthiazol-2-yl)-2,5-diphenyltetrazolium bromide or MTT) to purple formazan crystals by metabolically active cells [23]. Briefly, MDCK, A549 and DF-1 cells (4 × 10^4^ cells per well) were absorbed with 0.01 MOI of E01EE and E01GK at 37 °C for 1 h. Cells were then washed with PBS to remove the virus and incubated for another 24, 48, 72 or 96 h. Afterwards, 50 μL/well of MTT solution (3 mg/mL) was added and incubated at 37 °C for 4 h. Following centrifugation, the plate was added with 100 μL/well of DMSO and incubated at room temperature for 30 min. Optical density value was measured at 540 nm using an ELISA reader (Sunrise^TM^, Männedorf, Switzerland), and cell relative viability was calculated [23]. Mock-treated cells served as control whose viability was regarded as 100% at each observation time point.

### 2.6. Chloramphenicol Acetyl Transferase Enzyme-Linked Immunosorbent Assay (CAT ELISA)

Influenza ribonucleoprotein (RNP) activity was measured using the CAT ELISA kit (Roche, Indianapolis, IN, USA). The RNP (ribonucleoprotein) complex was composed of PB1, PB2, PA and NP proteins. First, the RNP coding sequences derived from E01EE and E01GK, including PA, NP, PB1 739E, PB1 739G, PB2 627E and PB2 627K, were cloned into plasmid pcDNA3.1 (Thermo). HEK 293T cells (ATCC) were then cotransfected with 1 μg of the various RNP components cloned into pcDNA3.1. A plasmid pPOLI-CAT-RT kindly from Chang Gung University (Taoyuan, Taiwan) containing a reporter gene (CAT) flanked by the viral promoters was concurrently transfected into the cells. Plasmid pcDNA3.1/CAT (Thermo) was used as the positive control. The total cell lysate was extracted 48 h after transfection using the 1× Lysis Buffer provided in the kit. After quantifying the protein content, each protein sample was diluted to 100 ng/mL using Sample Buffer. The sample CAT levels were subsequently determined according to the manufacturer’s instructions.

### 2.7. Animals

Five-week-old specific-pathogen-free (SPF) balb/c mice (Bio Lasco, Taipei, Taiwan) were used in this study. The animal experiment was approved by Institutional Animal Care and Use Committee of National Taiwan University with the project identification code NTU105-EL-00140, which was approved on 27 March 2019. Groups of six mice were anesthetized with isoflurane (Rhodia Organique Fine, Bristol, UK) and inoculated intranasally with 10^4^ TCID_50_/mL of E01EE, 10^4^ TCID_50_/mL of E01GK, 10^6^ TCID_50_/mL of E01GK or PBS in a 50 μL volume (25 μL per nostril). The clinical signs and the body weights were monitored every day. The animals were anesthetized with isoflurane at 4, 8, 11 and 15 d.p.i. (days post inoculation). Twenty-five μL of PBS was instilled into each nostril, pipetted three times, and all of the nasal wash liquid was drawn back. Oropharyngeal and anal swab samples were also taken. Three mice from each group were euthanized with CO_2_ at 4 and 15 d.p.i. The heart blood was collected for virus detection. The lungs, tracheas and nasal turbinates were collected for histopathological and immunohistochemistry assay. Oropharyngeal and anal swab samples were together dissolved in 200 μL of PBS and mixed with the nasal washes. Viral RNA from the mixed samples was extracted using Viral Nucleic Acid Extraction Kit (Geneaid Biotech, Taipei, Taiwan). The RNA from 200 μL of blood samples was also extracted using the same kit. All RNA was stored at −80 °C until use.

### 2.8. Quantitative Reverse Transcription Real Time PCR (qRT-PCR)

To quantify the viral RNA copies in the animal samples, qRT-PCR was performed using the modified primers and probe targeting nucleoprotein gene [24] and the RT-qPCR-probe 1-step Go No-ROX kit (Biosystems, London, UK). The reaction was conducted using qTOWER3G Real-time PCR Thermal Cycler (Analytik Jena AG, Jena, Germany). For each qRT-PCR reaction, 5 μL of RNA was used as template in a final volume of 20 μL. The RNA standard from known copy numbers was prepared as described [25], and a standard curve (linear trend line) corresponding to the qRT-PCR ct value was obtained. The viral RNA amount from the mouse samples was acquired by comparing to the standard curve. Interestingly, a standard curve (polynomial trend line, log_10_ of copy number vs. peak S/N value) could also be achieved while employing one-step RT-PCR (QIAGEN, Hilden, Germany) and Qsep100 capillary electrophoresis (BiOptic, New Taipei City, Taiwan), and the viral RNA amount was obtained which was almost the same as those obtained from qRT-PCR.

### 2.9. Histopathological Analysis and Immunohistochemistry (IHC)

Four μm thick Formalin-fixed paraffin-embedded (FFPE) tissue slides were deparaffinized with xylene, rehydrated with decreasing concentrations of ethanol, and rinsed with TBST (Tris-buffered saline, 0.1% Tween 20). The slides were processed using hematoxylin and eosin (H&E) staining for histopathological analysis. For the IHC staining, antigen retrieval was applied by boiling the slides in the EDTA-based retrieval buffer (Trilogy^TM^) (Cell Marque, CA, USA) in a microwave (EZ-Retriever^®^ System, BioGenex Laboratories, San Ramon, CA, USA) at 95 °C for 10 min. The slides were then incubated with 2.5% normal goat serum (Dako, CA, USA) diluted in PBS (pH 7.4) at room temperature for 30 min to block non-specific binding sites. Antigen expression was detected using hyperimmune chicken serum, derived from chickens immunized with the A/Chicken/Taiwan/2838v/00 virus, at 1:800 dilution in 2.5% normal goat serum for 1 h at room temperature. Endogenous peroxidase activity was blocked with 3% hydrogen peroxide (KYB, New Taipei City, Taiwan) for 15 min. Afterward, the slides were then treated with HRP conjugated goat anti-chicken IgG (H+L)(SeraCare, Milford, MA) (1:1000 in dilution) for 40 min at room temperature. After washing in TBST, the reaction products were visualized with 2% diaminobenzidine (Dako, Glostrup, Denmark) for 3 min at room temperature. The slides were finally counterstained with Mayer’s hematoxylin solution (Muto Pure Chemicals, Tokyo, Japan) for 40 s. 

### 2.10. Histopathological and Immunohistochemistry Scoring

The lung tissue histopathological scoring was given a ranked score of 0–4 according to the interstitial pneumonia severity: 0 = normal, 1 = mild interstitial pneumonia, 2 = moderate multifocal interstitial pneumonia, 3 = moderate diffuse interstitial pneumonia, 4 = severe interstitial pneumonia. The IHC scoring was given based on the average antigen amount. Positive cells were counted in five random 200× fields for each tissue section and the average was taken. A ranked score of 0–4 was given as an estimate of the positive cell numbers in tissue from each FFPE block: 0 = no influenza antigen-positive cells, 1 = 1–10 positive cells, 2 = 11–30 positive cells, 3 = 31–100 positive cells, and 4 ≥ 100 positive cells.

### 2.11. Statistical Analyses

All statistically analyses were performed using GraphPad Prism 6.0 (GraphPad Software, San Diego, CA, USA). Statistically significant differences among groups were determined using analysis of variance (ANOVA). The *p* value of <0.05 was considered statistically significant. 

## 3. Results

### 3.1. Viral Dynamics and Plaque Assay

**Figure 1:**
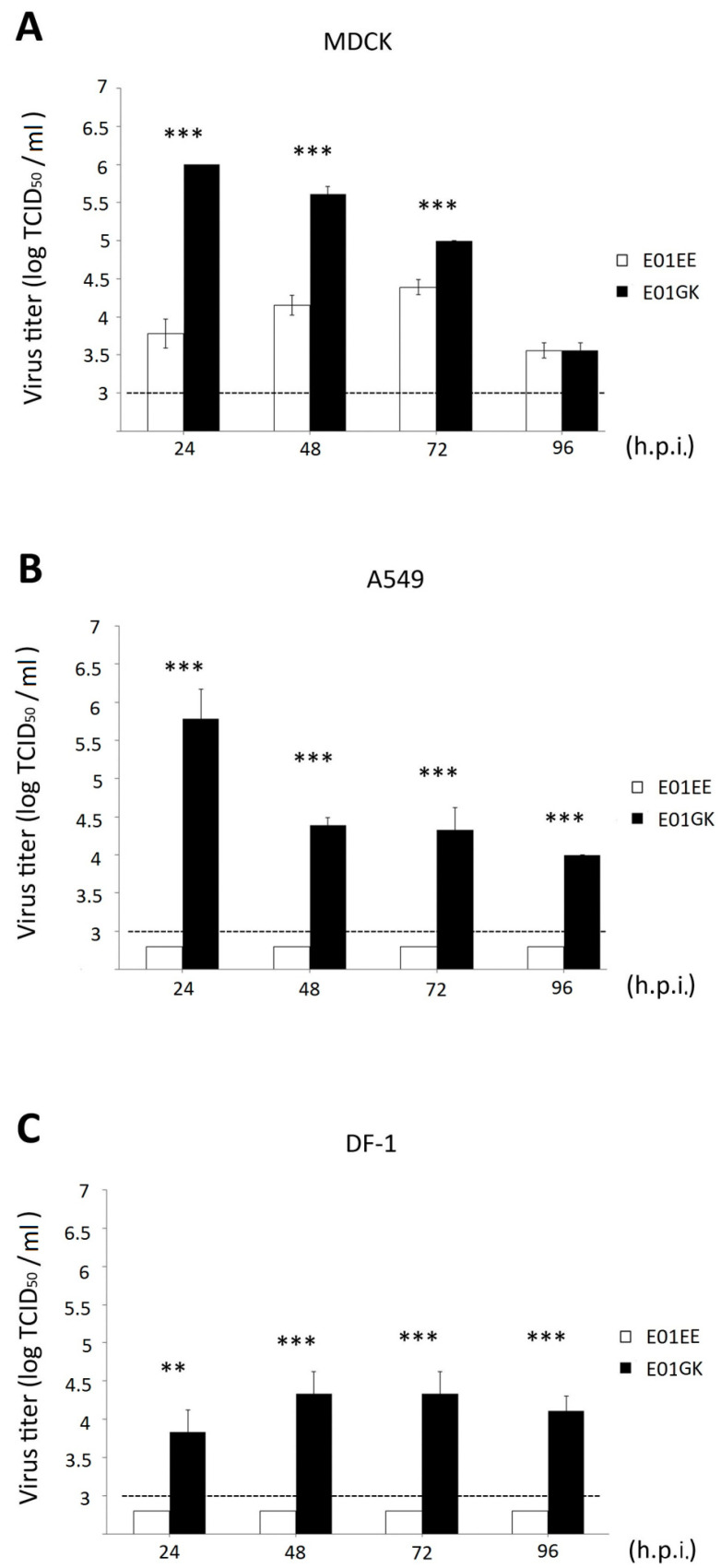
The viral dynamics of Madin–Darby canine kidney (MDCK) (**A**), A549 (**B**) and DF-1 cells (**C**). The cells were infected with 0.01 MOI of viruses for 1 h. The culture supernatants were collected at 24-h intervals until 96 h for viral TCID_50_ measurement on MDCK cells. The lowest detectable titer is 10^3^ TCID_50_/mL. h.p.i.: hours post infection. **: *p* < 0.01, ***: *p* < 0.001.

**Figure 2:**
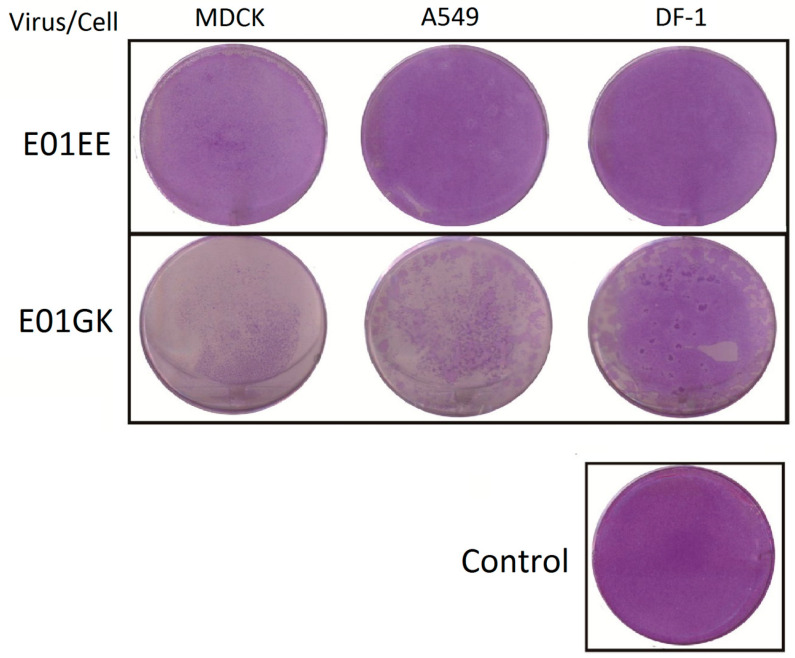
The comparison of plaque formation. MDCK, A549 and DF-1 cells were infected with 0.01 MOI of viruses for 1 h and the culture supernatants at 24 h.p.i. were collected for plaque assay on MDCK cells.

E01EE was obtained from one egg passage of E01, possessing PB1 739E and PB2 627E. E01GK was gotten from two MDCK cell passages of E01EE, having PB1 739G and PB2 627K. Purified E01EE and E01GK were acquired after three times of plaque purifications (Table 1). Confluent MDCK, A549 and DF-1 cells were absorbed with 0.01 MOI of E01EE and E01GK at 37 °C for 1 h and incubated for another 96 h after removing the viruses. The culture supernatants were collected at 24-h intervals. Plaque assay and virus titration were performed on MDCK cells [22]. E01GK showed significantly higher virus titer than E01EE in all infected cells over the 96-h observation, indicating the higher viral infection efficiency of E01GK (Figure 1). The E01GK highest titer at 24 h.p.i. in MDCK and A549 cells could reach around 10^6^ TCID_50_/mL. However, E01EE only showed low replication in MDCK cells, and no detectable titers (below 10^3^ TCID_50_/mL) were obtained in A549 and DF-1 cells over the 96-h course (Figure 1). Figure 2 compared the plaque forming condition on MDCK cells of E01EE and E01GK from the culture supernatants of infected MDCK, A549 and DF-1 cells at 24 h.p.i., and E01GK showed compellingly notable plaques. These results implied that E01GK had better infectivity than E01EE in mammalian and avian cells.

### 3.2. Cellular Viability Comparison

**Figure 3:**
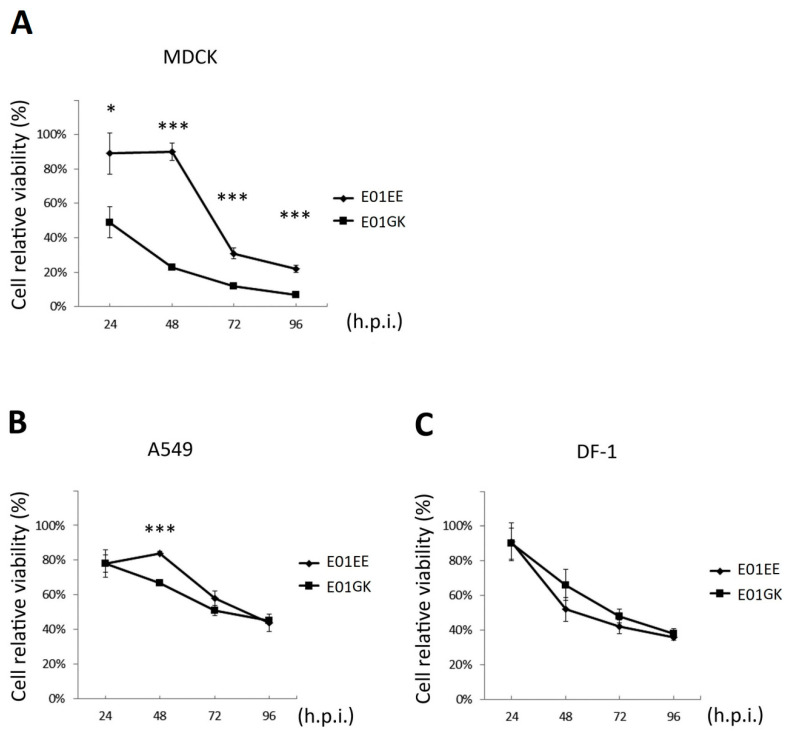
The relative viability of the virus infected cells was quantified using 3-(4,5-dimethylthiazol-2-yl)-2,5-diphenyltetrazolium bromide (MTT) assay. MDCK, A549 and DF-1 cells were absorbed with 0.01 multiplicity of infection (MOI) of E01EE and E01GK for 1 h and incubated for another 96 h. The cellular viability was compared with the mock-treat cells whose viability was regarded as 100% at each time point. (**A**) MDCK cells; (**B**) A549 cells; (**C**) DF-1 cells. h.p.i.: hours post infection. *: *p* < 0.05; ***: *p* < 0.001.

Cellular relative viability was measured using MTT assay. MDCK, A549 and DF-1 cells were absorbed with 0.01 MOI of E01EE and E01GK for 1 h and incubated for another 96 h. The MTT assay results showed that both E01EE and E01GK decreased cell relative viability in all kinds of infected cells over the time course comparing to the mock-treated cells whose viability was regarded as 100%. The result indicated the potential of dog H6N1 virus for interspecies transmission. E01GK caused greater cellular harm than E01EE over the whole 96 h in MDCK cells and in the first 48 h in A549 cells. However, in DF-1 cells, there were no significant differences between the two viruses (Figure 3). At 24 d.p.i., E01GK could rapidly cut the cellular viability down to half in MDCK cells (Figure 3A). The results indicated that E01GK induced more cellular death than E01EE, especially in MDCK cells. 

### 3.3. Viral Ribonucleoprotein (RNP) Activity Test

**Figure 4:**
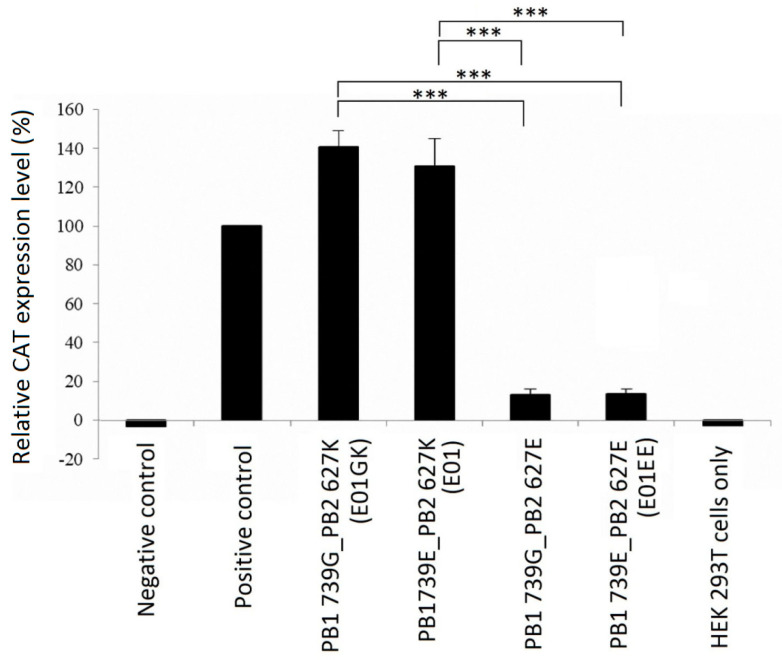
The viral ribonucleoprotein (RNP) activity was signaled using CAT ELISA and displayed as relative CAT expression level. All RNP complex varieties exhibited different combinations of PB1 and PB2 residues were tested. Negative control: plasmid pcDNA3.1 only; Positive control: plasmid pcDNA3.1/CAT. No significant differences were noted between E01GK and E01. There were also no significant differences among groups of negative control, PB1739G_PB2 627E, E01EE and HEK 293T cells. ***: *p* < 0.001.

The E01EE and E01GK sequence analysis results were shown in Supplement Table S1. There were total four nucleotide differences. However, only two amino acids were altered, which were PB1 739 and PB2 627. To assess the impact of the altered amino acids on viral transcription, RNP activity was tested using CAT ELISA kit. HEK 293T cells were cotransfected with various RNP component genes cloned into pcDNA3.1 for 48 h. Plasmid pcDNA3.1/CAT was employed as the positive control. The higher CAT level indicated higher viral RNA polymerase activity. The CAT ELISA results showed that PB2 627 was the crucial determinant of RNP activity but not the PB1 739 residue (Figure 4). Regardless what the PB1 729 residue was, once the influenza virus had the PB2 E627K mutation, the RNP activity could increase dramatically. This may account for the high pathogenicity of E01GK, which was consistent with the previous studies [26,27].

### 3.4. The Virus Infection of Mice

**Figure 5:**
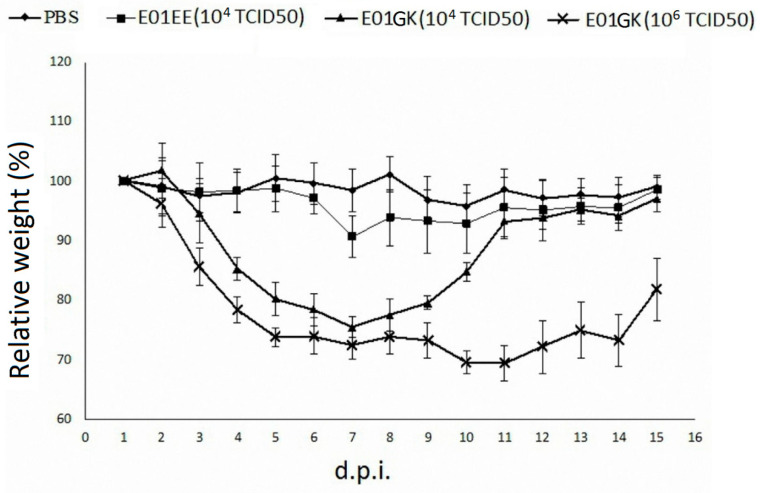
The body weight changes in mice. The values represent the average body weights compared to the baseline weight ± standard deviations from the mice in the same group. Mice were inoculated intranasally with 10^4^ TCID_50_/mL of E01EE, 10^4^ TCID_50_/mL of E01GK, 10^6^ TCID_50_/mL of E01GK or PBS in a 50 μL volume (25 μL per nostril). d.p.i.: days post inoculation.

Since the E01EE and E01GK influenza A viruses were from a dog isolate, whether the viruses had infectivity in mice was examined. Six mice per group were inoculated with 10^4^ TCID_50_/mL of E01EE, 10^4^ TCID_50_/mL of E01GK, 10^6^ TCID_50_/mL of E01GK or PBS in a 50 μL volume. Interestingly, regardless of what viruses or dosages were used, all virus-inoculated mice displayed lethargy from the 3rd day after inoculation. The E01EE group body weight showed a mild decrease from 5 d.p.i. to 7 d.p.i. and gradually recovered. Both E01GK dosage groups showed notable body weight decrease from 3 d.p.i. to 7 d.p.i. Afterwards the 10^4^ TCID_50_ group body weight slowly recovered but the 10^6^ TCID_50_ group remained low around 70–80% until euthanasia at 15 d.p.i. These results indicated that the dog isolated H6N1 influenza A viruses were able to cause illness in mice, and E01GK had a more severe impact than E01EE, especially at high dosage (Figure 5).

### 3.5. Virus Amount Quantification 

**Figure 6:**
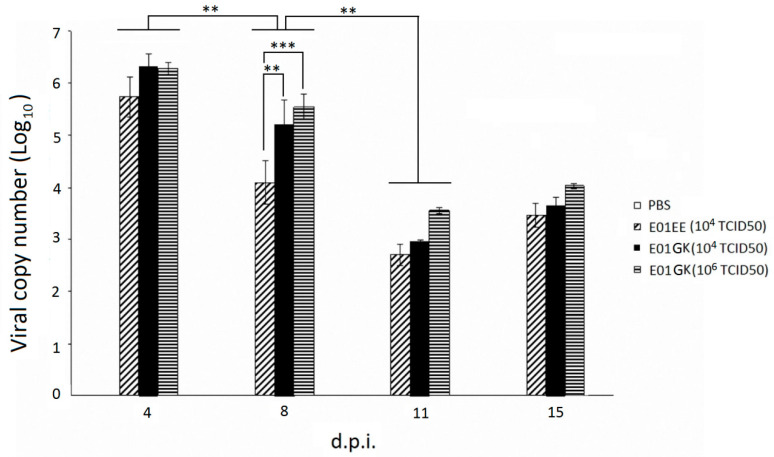
The virus quantification from swab and nasal wash samples using qRT-PCR. Mice were inoculated intranasally with 10^4^ TCID_50_/mL of E01EE, 10^4^ TCID_50_/mL of E01GK, 10^6^ TCID_50_/mL of E01GK or PBS in a 50 μL volume (25 μL per nostril). No detectable signals were noted in the negative PBS control group. d.p.i.: days post inoculation. **: *p* < 0.01; ***: *p* < 0.001.

The mouse sample virus amount was quantified using qRT-PCR. Figure 6 shows the virus shedding amount from the mixed swab and nasal wash samples. The viral copy numbers were around 10^6^ copy numbers at 4 d.p.i. in all experimental groups and gradually decreased to 10^3^–10^4^ at 11–15 d.p.i. The result indicated that the dog H6N1 could cause substantial virus shedding in mice in the first few infection days. There were significant differences between 4 and 8 d.p.i. (*p* < 0.01) and between 8 and 11 d.p.i. (*p* < 0.01) in all experimental groups but no significant differences (*p* > 0.05) between 11 and 15 d.p.i. among all groups (Figure 6). Interestingly, the E01EE group shedding viral number was significantly lower than 10^4^ TCID_50_ of E01GK group (*p* < 0.01) and 10^6^ TCID_50_ of E01GK group (*p* < 0.001) at 8 d.p.i.; this day was also consistent with the biggest body weight gap (Figure 5). The virus amount in blood samples was kept at a low range from 10^2.5^ to 10^3^ copy numbers in all groups at 4 d.p.i. and 15 d.p.i., and no significant differences among the virus groups and between the two days (Supplement Figure S1). No detectable signals were noted in the PBS negative control groups in all mouse samples.

### 3.6. Histopathological and Immunohistochemistry (IHC) Evaluation

**Figure 7:**
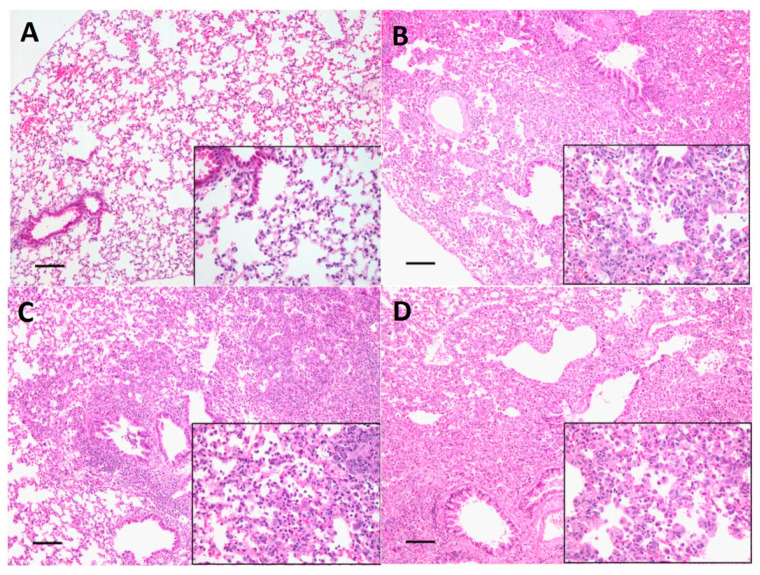
H&E staining of mice inoculated with dog H6N1 at 15 d.p.i. and the histopathological evaluation of pathogenicities in lung tissue caused by PBS (**A**), 10^4^ TCID_50_/mL of E01EE (**B**), 10^4^ TCID_50_/mL of E01GK (**C**) and 10^6^ TCID_50_/mL of E01GK (**D**). The scale bar size is 100 μm for the left 100× magnification field. The right down corner is 400× magnification field.

**Figure 8:**
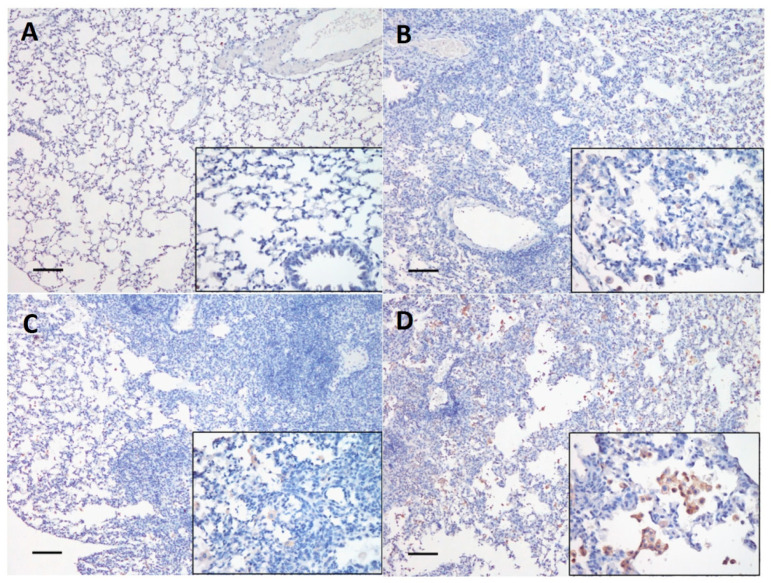
H6N1 viral antigen detection in mouse lung tissue at 15 d.p.i. using IHC staining. The mice were inoculated with PBS (**A**), 10^4^ TCID_50_/mL of E01EE (**B**), 10^4^ TCID_50_/mL of E01GK (**C**) and 10^6^ TCID_50_/mL of E01GK (**D**). The scale bar is 100 μm for the left 100× magnification field. The right down corner is 400× magnification field.

Dog H6N1 virus-inoculated mice showed lesions and influenza antigen-positive cells in lungs at 4 and 15 d.p.i. The severity was more obvious at 15 d.p.i. than 4 d.p.i. Table 2 shows the histopathological and IHC scoring results at 15 d.p.i. The mouse lung tissue histopathological evaluation was analyzed using H&E staining. While mice in the PBS negative control showed no lesions, mice in all virus inoculated groups at 15 d.p.i. showed influenza associated lesions from multifocal infiltration of numbers of cells, predominantly macrophages, type II pneumocytes, and lymphocytes in the interstitium and lung airspaces (Figure 7). Compared with mice in the E01EE group, more severe and diffuse interstitial pneumonia distribution was noted in mice inoculated with either dosage of the E01GK groups (Table 2). Furthermore, while no antigen positive cells were identified in mice in PBS control group, variable numbers of influenza antigen-positive cells, characterized by intracytoplasmic positivity in the macrophages and hyperplastic type II pneumocytes, were identified in the lungs of all virus infected groups at 15 d.p.i. based on the IHC evaluation (Figure 8). Among all virus infected groups, the 10^6^ TCID_50_ E01GK group had the largest number of positive cells (Table 2). Interestingly, there were not perceivable lesions or influenza antigen-positive cells in mouse tracheal and nasal turbinate tissues, neither at 4 d.p.i. nor at 15 d.p.i. 

## 4. Discussion

The H6N1 avian influenza A viruses have been endemic in chickens in Taiwan over 45 years and have established a unique lineage which differs from those circulating in Hong Kong and in Southeastern China [13,28,29]. A previous study indicated that it would cause respiratory distress, decreased egg production or increased flock mortality in poultry [13]. Unlike avian species, H6 subtype infection in mammals is rare. Beare and Webster reported that humans who were inoculated with avian-origin H6N1 virus shed the virus and exhibited mild clinical symptoms but would not produce a detectable antibody response [30]. Some chicken H6N1 viruses in Taiwan have acquired a G228S substitution in viral HA protein since 2000 [14]. H6N1 viruses with HA G228S substitution have become predominant in poultry after 2005 [14,29]. The HA G228S substitution was considered to contribute to HA protein specificity for the human SAα2-6Gal receptors [31,32]. Until 2013, the world’s first human H6N1 infection case was demonstrated in Taiwan [14]. The dog H6N1 natural infection case was also confirmed in 2014 [15]. Both the human and dog H6N1 viruses were of avian origin and had HA G228S substitution, which might be a factor enabling the viral infection in mammals [14,15]. Furthermore, the C-terminal PDZ domain ligand (PL) of avian influenza virus NS1 protein was found virulent in a mouse model [33,34]. Both E01EE and E01GK in this study harbored the HA G228S substitution and NS1 PL with EPEV residues [14], which might account for their infectivity in mice. Interestingly, the PB2 627 position was E in human A/Taiwan/2/2013 virus but was K in dog E01 parental and E01GK progeny viruses. The PB2 E627K substitution could contribute viral replication ability in hosts and was regarded the best characterized mammalian adaptation [32].

The E01 was isolated from a 4-month-old stray dog co-infected with canine distemper virus. The dog was rescued to hospital and died 19 days later. It exhibited purulent nasal discharge, cough, fever and showed pneumonia on radiographs. Necropsy was declined by the owner. Further information was unavailable. Therefore, we could not deduce whether the virus accidentally infected the dog or had adapted to the dog. However, based on the molecular biology evidence, we conclude that the adaptation might have occurred since the virus possessed PB2 E627K mutation besides HA G228S substitution.

Viral coat HA protein binding to sialylated glycan receptors on host epithelial cells is the critical initial step in influenza A viral infection and transmission. The important factor of potential infectivity of influenza A viruses is the receptor binding site affinity for the host [35]. A switch in the HA binding specificity from SAα2-3Gal linked to SAα2-6Gal linked glycans is essential for the crossover of the viruses from avian to human hosts [36,37]. The glycan receptor types in dog trachea and lung had been studied. Both SAα2-3Gal and SAα2-6Gal receptors were in epithelial cells of dog tracheas and bronchi/bronchioles. However, only SAα2-6Gal receptors were shown in dog lung alveolar cells [35]. In mice, only SAα2-3Gal receptors are expressed in the trachea and only SAα2-6Gal receptors are distributed in the pulmonary parenchyma [38]. This may explain why this avian-origin dog H6N1 virus could infect dogs and mice and its potential interspecies transmission role among avian species, mammals or humans. This study found that both E01EE and E01GK caused weight loss and pneumonia in mice. However, no apparent lesions were noted in tracheal and nasal turbinate tissues. We therefore speculated that the dog H6N1 virus might have higher affinity toward SAα2-6Gal receptors; nevertheless, further studies are still needed.

A previous study has proven that amino acid alterations could change viral adaptation to tissue culture [39]. Virulent viruses could be attenuated via serial cell culture passages [40]. In this study we demonstrated amino acid alterations over passages through different culture systems. The PB2 627 position of A/canine/Taiwan/E01/2014 (E01) was K but was altered into E (E01EE) through one embryonated egg passage. It suggests that PB2 K627E mutation resulted from an adaptation of the virus to the avian host. Following two MDCK cell passages, the residue was turned back into K (E01GK). E01EE and E01GK showed significant divergences in virus growth dynamics, cellular damage and the mouse pathogenicity. Although E01EE and E01GK were also different in PB1 739 (Table 1), it did not have significant influence on RNP activity (Figure 4). Whether the PB1 E739G mutation also arose from the virus adaptation to the mammalian host needs more studies to clarify.

The shedding virus amount from nasal–oral and anal samples reached around 10^6^ copy numbers at 4 d.p.i. in all experimental groups and gradually decreased until 11 d.p.i. Although at 15 d.p.i. the amount seemed to be backed a little, there was no statistical significance between 11 and 15 d.p.i. The decreased virus amount may associate with anti-viral antibodies production. In influenza A virus-infected mice, the antibodies generated by degrees and reached the peak at 8–10 d.p.i. for IgM and at 25 d.p.i. for IgG [41]. Interestingly, the blood sample virus amount was kept at a low range over 15 d.p.i., which was also barely detectable in agarose gel. This indicated that the dog H6N1was shed primarily from the upper respiratory tract or digestive tract but did not cause serious viremia in mice.

Based on the results from this study, we concluded that the avian sourced- dog H6N1 virus, A/canine/Taiwan/E01/2014, had notable infectivity in mammals. Both its progeny viruses E01EE and E01GK decreased cell relative viability in tested mammalian cells in addition to avian cells. They also caused lethargic signs, body weight loss, virus shedding and lung lesions in mice. We also proved that the viral virulence could be changed via host adaptation. Moreover, the PB2 E627K mutation was a key residue of the viral pathogenicity. However, comparison of avian, canine and human sourced H6N1 viruses in parallel would be worth further future study efforts. 

## Figures and Tables

**Table 1:** The amino acid divergences among A/canine/Taiwan/E01/2014 (E01) and its derivatives E01EE and E01GK.

Viruses	Amino Acid	
	PB1 Position 739	PB2 Position 627
E01	E	K
E01EE	E	E
E01GK	G	K

**Table 2:** Histopathological and immunohistochemistry scoring of the lesions and viral antigens from mouse lung tissue ^a^ at 15 d.p.i.

	PBS	10^4^ TCID_50_/mL of E01EE	10^4^ TCID_50_/mL of E01GK	10^6^ TCID_50_/mL of E01GK
H&E ^b^	0	2	3	3
IHC ^c^	0	1	2	3

^a^ No lesions and influenza antigen-positive cells were noted other than the lung tissue, including tracheas and nasal turbinates. ^b^ The histopathological scoring: 0 = normal, 1 = mild interstitial pneumonia, 2 = moderate multifocal interstitial pneumonia, 3 = moderate diffuse interstitial pneumonia, 4 = severe interstitial pneumonia. ^c^ The influenza antigen-positive cell numbers in 200× fields of each FFPE block based on IHC assay: 0 = no influenza antigen-positive cells, 1 = 1–10 positive cells, 2 = 11–30 positive cells, 3 = 31–100 positive cells, and 4 ≥ 100 positive cells.

## Supplementary Materials

The following are available online at https://www.mdpi.com/1999-4915/12/7/704/s1, Table S1: The sequence analysis results showing the different nucleotides and the resultant amino acids between E01EE and E01GK, Figure S1: The virus quantification from blood samples using qRT-PCR.

## References

[1] Lipatov A.S., Govorkova E.A., Webby R.J., Ozaki H., Peiris M., Guan Y., Poon L., Webster R.G. (2004). Influenza: Emergence and control. J. Virol..

[2] Crawford P.C., Dubovi E.J., Castleman W.L., Stephenson I., Gibbs E.P., Chen L., Smith C., Hill R.C., Ferro P., Pompey J. (2005). Transmission of equine influenza virus to dogs. Science.

[3] Song D., Kang B., Lee C., Jung K., Ha G., Kang D., Park S., Park B., Oh J. (2008). Transmission of avian influenza virus (H3N2) to dogs. Emerg. Infect. Dis..

[4] Payungporn S., Crawford P.C., Kouo T.S., Chen L.M., Pompey J., Castleman W.L., Dubovi E.J., Katz J.M., Donis R.O. (2008). Influenza A virus (H3N8) in dogs with respiratory disease, Florida. Emerg. Infect. Dis..

[5] Song D., Lee C., Kang B., Jung K., Oh T., Kim H., Park B., Oh J. (2009). Experimental infection of dogs with avian-origin canine influenza A virus (H3N2). Emerg. Infect. Dis..

[6] Li S., Shi Z., Jiao P., Zhang G., Zhong Z., Tian W., Long L.P., Cai Z., Zhu X., Liao M. (2010). Avian-origin H3N2 canine influenza A viruses in Southern China. Infect. Genet. Evol..

[7] Zhan G.J., Ling Z.S., Zhu Y.L., Jiang S.J., Xie Z.J. (2012). Genetic characterization of a novel influenza A virus H5N2 isolated from a dog in China. Vet. Microbiol..

[8] Song Q.Q., Zhang F.X., Liu J.J., Ling Z.S., Zhu Y.L., Jiang S.J., Xie Z.J. (2013). Dog to dog transmission of a novel influenza virus (H5N2) isolated from a canine. Vet. Microbiol..

[9] Bunpapong N., Nonthabenjawan N., Chaiwong S., Tangwangvivat R., Boonyapisitsopa S., Jairak W., Tuanudom R., Prakairungnamthip D., Suradhat S., Thanawongnuwech R. (2014). Genetic characterization of canine influenza A virus (H3N2) in Thailand. Virus Genes.

[10] Sun Y., Sun S., Ma J., Tan Y., Du L., Shen Y., Mu Q., Pu J., Lin D., Liu J. (2013). Identification and characterization of avian-origin H3N2 canine influenza viruses in northern China during 2009–2010. Virology.

[11] Songserm T., Amonsin A., Jam-on R., Sae-Heng N., Pariyothorn N., Payungporn S., Theamboonlers A., Chutinimitkul S., Thanawongnuwech R., Poovorawan Y. (2006). Fatal avian influenza A H5N1 in a dog. Emerg. Infect. Dis..

[12] Song D., Moon H.J., An D.J., Jeoung H.Y., Kim H., Yeom M.J., Hong M., Nam J.H., Park S.J., Park B.K. (2012). A novel reassortant canine H3N1 influenza virus between pandemic H1N1 and canine H3N2 influenza viruses in Korea. J. Gen. Virol..

[13] Lee M.S., Chang P.C., Shien J.H., Cheng M.C., Chen C.L., Shieh H.K. (2006). Genetic and pathogenic characterization of H6N1 avian influenza viruses isolated in Taiwan between 1972 and 2005. Avian Dis..

[14] Wei S.H., Yang J.R., Wu H.S., Chang M.C., Lin J.S., Lin C.Y., Liu Y.L., Lo Y.C., Yang C.H., Chuang J.H. (2013). Human infection with avian influenza A H6N1 virus: An epidemiological analysis. Lancet Respir. Med..

[15] Lin H.T., Wang C.H., Chueh L.L., Su B.L., Wang L.C. (2015). Influenza A(H6N1) Virus in Dogs, Taiwan. Emerg. Infect. Dis..

[16] Coloma R., Valpuesta J.M., Arranz R., Carrascosa J.L., Ortin J., Martin-Benito J. (2009). The structure of a biologically active influenza virus ribonucleoprotein complex. PLoS Pathog..

[17] DesRochers B.L., Chen R.E., Gounder A.P., Pinto A.K., Bricker T., Linton C.N., Rogers C.D., Williams G.D., Webby R.J., Boon A.C. (2016). Residues in the PB2 and PA genes contribute to the pathogenicity of avian H7N3 influenza A virus in DBA/2 mice. Virology.

[18] Cheng K., Yu Z., Chai H., Sun W., Xin Y., Zhang Q., Huang J., Zhang K., Li X., Yang S. (2014). PB2-E627K and PA-T97I substitutions enhance polymerase activity and confer a virulent phenotype to an H6N1 avian influenza virus in mice. Virology.

[19] Yu Z., Cheng K., Sun W., Zhang X., Li Y., Wang T., Wang H., Zhang Q., Xin Y., Xue L. (2015). A PB1 T296R substitution enhance polymerase activity and confer a virulent phenotype to a 2009 pandemic H1N1 influenza virus in mice. Virology.

[20] Chen G.W., Kuo S.M., Yang S.L., Gong Y.N., Hsiao M.R., Liu Y.C., Shih S.R., Tsao K.C. (2016). Genomic Signatures for Avian H7N9 Viruses Adapting to Humans. PLoS ONE.

[21] Wang C., Lee H.H., Yang Z.F., Mok C.K., Zhang Z. (2016). PB2-Q591K Mutation Determines the Pathogenicity of Avian H9N2 Influenza Viruses for Mammalian Species. PLoS ONE.

[22] WHO WHO Manual on Animal Influenza Diagnosis and Surveillance. https://apps.who.int/iris/handle/10665/68026.

[23] Shahsavandi S., Ebrahimi M.M., Mohammadi A., Zarrin Lebas N. (2013). Impact of chicken-origin cells on adaptation of a low pathogenic influenza virus. Cytotechnology.

[24] Zhang Z., Liu D., Sun W., Liu J., He L., Hu J., Gu M., Wang X., Liu X., Hu S. (2017). Multiplex one-step Real-time PCR by Taqman-MGB method for rapid detection of pan and H5 subtype avian influenza viruses. PLoS ONE.

[25] Wang L.C., Huang D., Chen H.W. (2016). Simultaneous subtyping and pathotyping of avian influenza viruses in chickens in Taiwan using reverse transcription loop-mediated isothermal amplification and microarray. J. Vet. Med. Sci..

[26] Kirui J., Bucci M.D., Poole D.S., Mehle A. (2014). Conserved features of the PB2 627 domain impact influenza virus polymerase function and replication. J. Virol..

[27] Chin A.W., Li O.T., Mok C.K., Ng M.K., Peiris M., Poon L.L. (2014). Influenza A viruses with different amino acid residues at PB2-627 display distinct replication properties in vitro and in vivo: Revealing the sequence plasticity of PB2-627 position. Virology.

[28] Lu Y.S., Sugimura T., Shieh H.K., Lee Y.L., Jong M.H. (1985). Isolation and identification of an influenza A virus in ducks in Taiwan. J. Chin. Soc. Vet. Med..

[29] Lee H.C., Hsu C.N., Kuo T.F., Wang C.H. (2005). Molecular epidemiology of avian influenza virus H6N1 in Taiwan from 2000 to 2003. Taiwan Vet. J..

[30] Beare A., Webster R. (1991). Replication of avian influenza viruses in humans. Arch. Virol..

[31] Stevens J., Blixt O., Tumpey T.M., Taubenberger J.K., Paulson J.C., Wilson I.A. (2006). Structure and receptor specificity of the hemagglutinin from an H5N1 influenza virus. Science.

[32] Schrauwen E.J., Fouchier R.A. (2014). Host adaptation and transmission of influenza A viruses in mammals. Emerg. Microbes. Infect..

[33] Jackson D., Hossain M.J., Hickman D., Perez D.R., Lamb R.A. (2008). A new influenza virus virulence determinant: The NS1 protein four C-terminal residues modulate pathogenicity. Proc. Natl. Acad. Sci. USA.

[34] Obenauer J.C., Denson J., Mehta P.K., Su X., Mukatira S., Finkelstein D.B., Xu X., Wan J., Ma J., Fan Y. (2006). Large-scale sequence analysis of avian influenza isolates. Science.

[35] Thongratsakul S., Suzuki Y., Hiramatsu H., Sakpuaram T., Sirinarumitr T., Poolkhet C., Moonjit P., Yodsheewan R., Songserm T. (2010). Avian and human influenza A virus receptors in trachea and lung of animals. Asian Pac. J. Allergy Immunol..

[36] Viswanathan K., Chandrasekaran A., Srinivasan A., Raman R., Sasisekharan V., Sasisekharan R. (2010). Glycans as receptors for influenza pathogenesis. Glycoconj. J..

[37] Suzuki Y. (2005). Sialobiology of influenza: Molecular mechanism of host range variation of influenza viruses. Biol. Pharm. Bull..

[38] Ning Z.Y., Luo M.Y., Qi W.B., Yu B., Jiao P.R., Liao M. (2009). Detection of expression of influenza virus receptors in tissues of BALB/c mice by histochemistry. Vet. Res. Commun..

[39] Van Loon A., de Haas N., Zeyda I., Mundt E. (2002). Alteration of amino acids in VP2 of very virulent infectious bursal disease virus results in tissue culture adaptation and attenuation in chickens. J. Gen. Virol..

[40] Song H., Santi N., Evensen O., Vakharia V.N. (2005). Molecular determinants of infectious pancreatic necrosis virus virulence and cell culture adaptation. J. Virol..

[41] Miao H., Hollenbaugh J.A., Zand M.S., Holden-Wiltse J., Mosmann T.R., Perelson A.S., Wu H., Topham D.J. (2010). Quantifying the early immune response and adaptive immune response kinetics in mice infected with influenza A virus. J. Virol..

