# Supplementary material for: Replication of a Dog-Origin H6N1 Influenza Virus in Cell Culture and Mice
Source: Viruses. 2020 Jun 30;12(7):704. doi: 10.3390/v12070704 (PMC7412498; doi:10.3390/v12070704)
Supplement: Supplementary file 1 [file viruses-12-00704-s001.pdf]

viruses

Supplementary File

Supplementary Table S1. The sequence analysis results showing the different nucleotides and the resultant amino acids between E01EE and E01GK.

Viruses	The nucleotides and the resultant amino acid[a]			
	PB1 position 78	PB1 position 218	PB1 position 739	PB2 position 627
E01EE	GAG (E)	TTA (L)	GAG (E)	GAG (E)
E01GK	GAA (E)	CTA (L)	GGG (G)	AAG (K)

[a]: E: Glutamic acid; L: Leucine; G: Glycine; K: Lysine

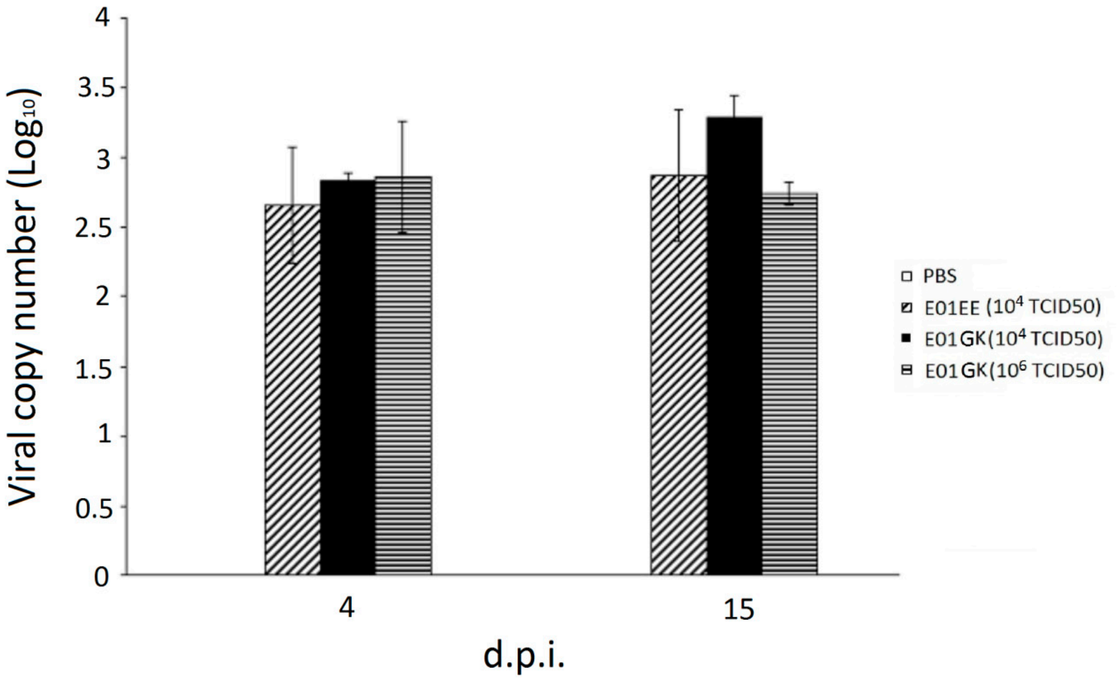

Supplementary Figure S1. The virus quantification from blood samples using qRT-PCR. No detectable signals were noted in the negative PBS control group. Mice were inoculated intranasally with 10^4 $TCID_{50}$/mL of E01EE, 10^4 $TCID_{50}$/mL of E01GK, 10^6 $TCID_{50}$/mL of E01GK or PBS in a 50 µL volume (25 µL per nostril). d.p.i.: days post inoculation.

© 2020 by the authors. Licensee MDPI, Basel, Switzerland. This article is an open access article distributed under the terms and conditions of the Creative Commons Attribution (CC BY) license (http://creativecommons.org/licenses/by/4.0/).
